# Mobility, electricity, and parking data for EV infrastructure in the Amsterdam Metropolitan Area

**DOI:** 10.1016/j.dib.2022.108270

**Published:** 2022-05-16

**Authors:** Pablo Muñoz Unceta, Bardia Mashhoodi

**Affiliations:** a Landscape Architecture and Spatial Planning Group, Department of Environmental Sciences, Wageningen University & Research, the Netherlands; and Researcher. Fab Lab Barcelona. Institute for Advanced Architecture of Catalonia, Barcelona, Spain; b Landscape Architecture and Spatial Planning Group, Department of Environmental Sciences, Wageningen University & Research, the Netherlands

**Keywords:** Mobility, Parking capacity, Electricity demand, GIS, Amsterdam Metropolitan Area

## Abstract

The dataset combines and aggregate five data types of the Amsterdam Metropolitan Area in a polygon GIS shapefile with 79 three-digit postcode areas, and a csv file, including the outcome of an origin-destination analysis. The dataset area includes 1661 residential zones (“wijken”, in the terminology used by the Dutch Central Bureau for Statistics) of the Amsterdam Metropolitan Area (AMA), and the data aggregated was originally generated between 2017 and 2019. The first dataset, in format of GIS shapefile, contains data on mobility, parking capacity, electricity demand, census data and cadaster data. The second dataset, csv format, includes the outcomes of an origin-destination analysis between the 79 three-digit postcodes areas in AMA. The two datasets provide the basis for analysis and adaptation of electric mobility in AMA.

## Specifications Table

**Table:** 

Subject	Planning and Development
Specific subject area	Metropolitan and Electric Mobility
Type of data	Georeferenced datasets in the format of GIS polygon shapefile, and data in a csv file
How the data were acquired	The raw data is acquired from Open Street Map, the Statistical Bureau of the Netherlands, the Dutch Cadaster, and the Dutch Ministry of Infrastructure and Water Management. The data, subsequently, is analyzed by use of Arc GIS Pro software.
Data format	Raw and analyzed (shapefile and csv)
Description of data collection	The data is collected from online public, open data sources and subsequently analyzed by use of Arc GIS Pro software.
Data source location	*•* Institution: Landscape Architecture and Spatial Planning Group, Department of Environmental Sciences, Wageningen University & Research, the Netherlands: https://www.wur.nl/en/Research-Results/Chair-groups/Environmental-Sciences/Landscape-Architecture-and-Spatial-Planning-1.htm *•* City: Wageningen *•* Country: the Netherlands *•* Latitude and longitude for collected data: The Netherlands (52°22′N 4°53′E) Institutions providing primary data and links: Statistics Netherlands (CBS): https://www.cbs.nl/ and https://www.pdok.nl/ Dutch Ministry of Infrastructure and Water Management: https://www.rijkswaterstaat.nl/wegen/wetten-regels-en-vergunningen/verkeerswetten/maximumsnelheid The Netherlands Cadaster: https://www.kadaster.nl/ Geofabrik and Open Street Map: http://download.geofabrik.de/
Data accessibility	Repository name: Mendeley Data Data identification number: DOI:10.17632/v4875253hz.1 Direct URL to data: https://data.mendeley.com/datasets/v4875253hz/1 Instructions for accessing these data: Open-access downloadable data. It includes a csv file and a folder with the GIS shapefile.

## Value of the Data

• The dataset brings different datasets together on a three-digit postcode scale, including data on different formats and sources.
• Researchers in transport, mobility and urban planning can benefit from the data.
• The data can be further used for regional and metropolitan studies on the use of electric vehicles and the implementation of new electricity-based mobility infrastructure. The dataset can facilitate research on energy transition.

## 1 Data Description

This article provides two datasets:

- A GIS shapefile with 79 three-digit postcode areas
- A csv file, including the outcome of an origin-destination analysis

Datasets are built from five distinct types of data: (1) Spatial data, (2) electricity consumption data, (3) mobility data, (4) census data and (5) cadaster data. To do so, the following datasets are collected, processed, and aggregated:

• Spatial data: Open Street Map provides GIS shapefiles for the Noord Holland province [1], including the road network in GIS vector shapefile format (gis_osm_roads_free_1) and parking spaces in GIS polygon shapefile format (gis_osm_traffic_a_free_1).
• Mobility data: Average speeds are extracted from the Dutch Traffic Rules and Traffic Signs Regulations 1990 [2], including the latest regulation on speed limits for highways [3].
• CBS Electricity Consumption: the statistical bureau of the Netherlands publishes data over energy consumption by postcode, differentiated in households’ and businesses’ demand [4].
• CBS Wijk 2019 census data: the statistical bureau of the Netherlands publishes data over the residential zones (Wijk) in 2019, in GIS polygon shape format. This data includes postcodes, number of inhabitants, households, and cars registered in each Wijk. Data is downloaded from the Maps Public Services Platform (PDOK) [5].
• Cadaster data: The Netherlands cadaster published building data, including building function, location, geometry, and age, among other attributes, for 2017, in GIS polygon and point shapefile format [6].

**Fig. 1:**
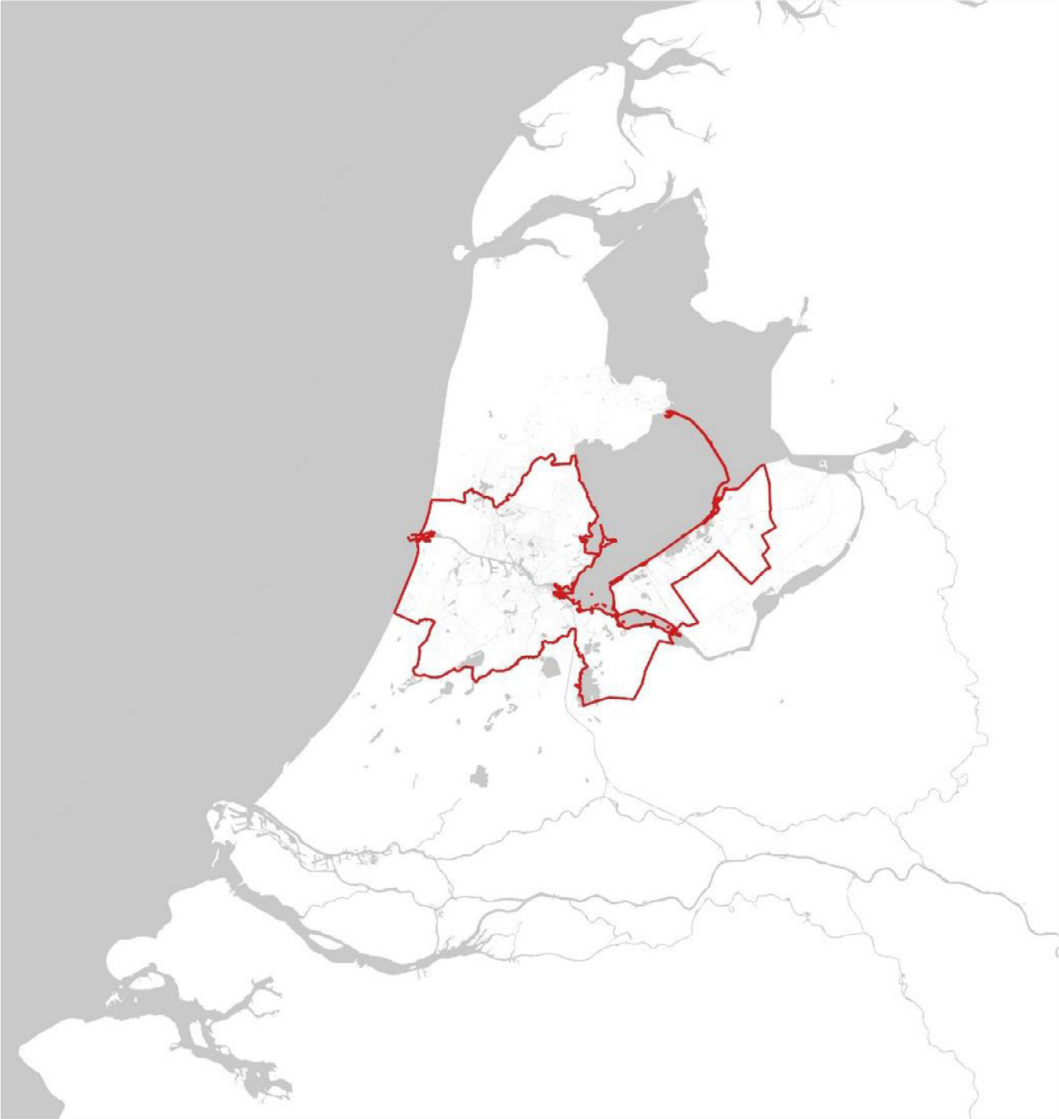
The location of the dataset area (AMA) in the Netherlands.

All datasets are collected at different scales. To aggregate them, data is delimited and trimmed to the AMA (Fig. 1).

The data is transformed in two datasets through a series of operations. In the following sections the collection and preparation of the raw data, the spatial analysis of them, and its aggregation in two datasets are explained. After these three sections, the potential uses of the obtained dataset are discussed.

## 2 Raw Data Collection

**Fig. 2:**
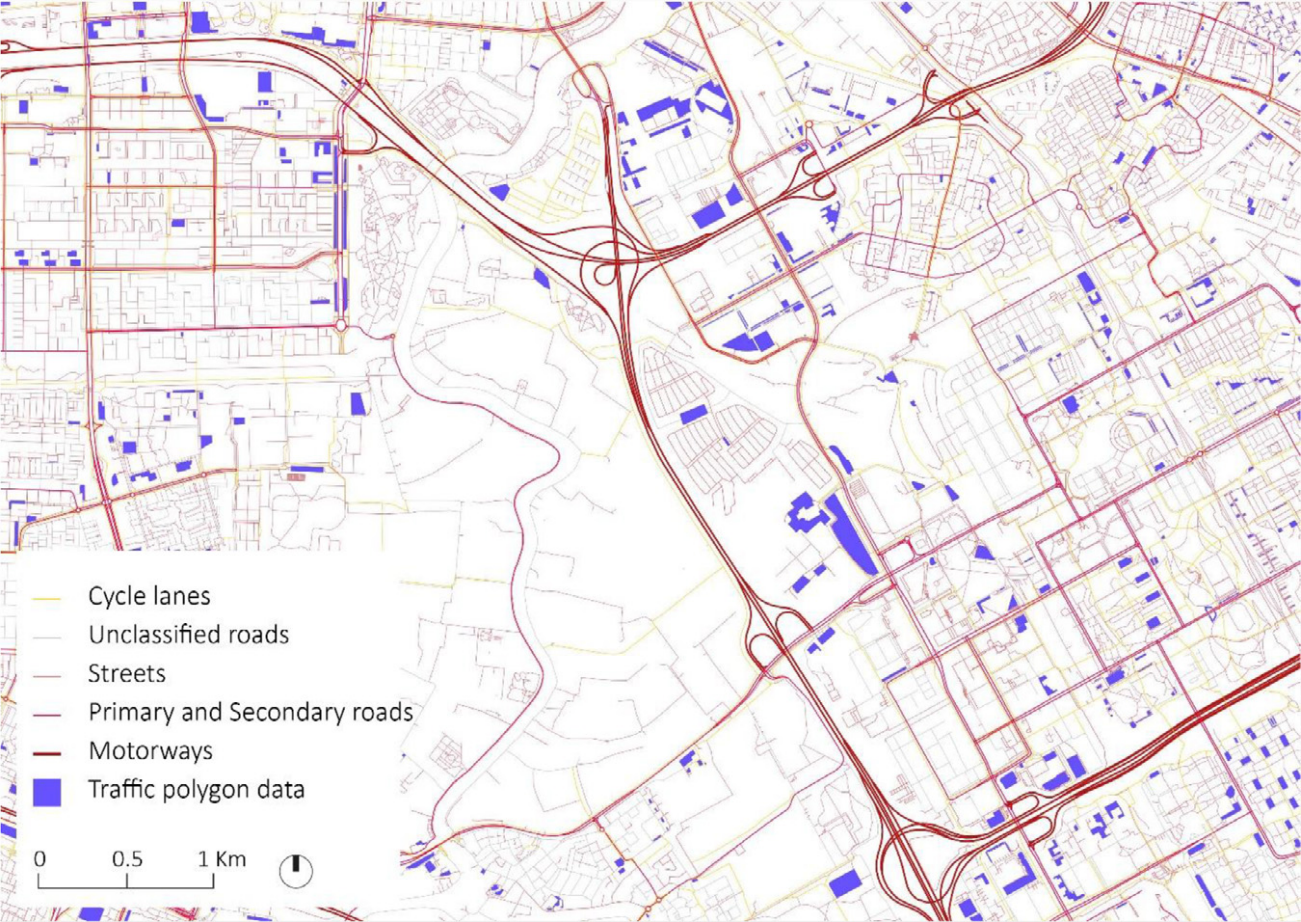
Zoom-in visualization of spatial data downloaded from Open Street Maps. Roads classified by type according to legend. Traffic polygon data includes parking sites, piers, fuel stations and other traffic data.
• Dutch Traffic Rules and Traffic Signs Regulations 1990 [2], and regulation on speed limits for highways from 2020 [3]: These regulations include the maximum car speed according to each road type. As cars do not necessarily travel at the maximum speed, an average speed of 75% maximum speed is considered (Table 1). This information can be joint to the road vector shapefile downloaded from Open Street Map using the attribute “fclass"^2^.
• Central Statistics Bureau of the Netherlands (CBS): Two datasets are downloaded. The first dataset [5] includes a GIS polygon shapefile of the residential zones ("wijk”) in the Netherlands in 2019, including socioeconomic census data over the zones. The second dataset [4] includes average energy consumptions data, differentiated by companies and households, and the types of energy (gas and electricity) in 2018. The data is aggregated by postcodes. Data is downloaded as a csv file.

Raw data included spatial, mobility, census, cadaster, and electricity consumption data. It was downloaded from four sources:

• Open Street Map [1]: A data set with all GIS shapefiles available for the Noord Holland province is downloaded through the website Geofabrik^1^ (Fig. 2). Two GIS shapefiles are selected to work with. First, a vector shapefile called “gis_osm_roads_free_1” with information on roads. This shapefile includes vectors for all roads and streets in the area, including attributes such as type of road (“fclass”). This shapefile includes also maximum speed information for each road. Nevertheless, this information is available for only 28.22% of the features. Second, a polygon shapefile called “gis_osm_traffic_a_free_1” with diverse information on traffic-related polygons, including parking sites, piers, fuel stations, etc., is used.

**Table 1:** Maximum speed by type of road according to Dutch regulation and average speed assumed for the case study.

Type of road	Maximum car speed (km per hour)	Average car speed (km per hour)
Streets in urban areas	50	37.5
High speed motorways	130	75
Motorways	100	75
Other roads	80	60
• Basic Register of addresses and buildings (“Basisregistratie Adressen en Gebouwen” in Dutch) [6]: Cadaster data is downloaded, including two datasets. One includes a GIS polygon shapefile with spatial and geometric data for all buildings in the Netherlands (“BAG 3D”). The second dataset includes a GIS point shapefile with data on the number of addresses (“BAG Adres”).

## 3 Spatial Analyses

Employing spatial analysis techniques, the original raw data are aggregated into two datasets: a GIS shapefile with data aggregated on three-digit postcode (PC3) areas, and a csv file with the result data of an Origin and Destination network analysis. Below, the steps taken in the spatial analysis for data aggregation are explained and elaborated.

### 3.1 Dataset area delimitation

The selected dataset area is AMA. The AMA is one of the five top economic regions in Europe, with a population of 2.5 million inhabitants, includes key infrastructure, such as the Schiphol airport and the port of Amsterdam, multiple universities, business districts, a media park, and a considerable concentration of tourism and leisure activities [7]. The border of the AMA includes 32 municipalities.

**Fig. 3:**
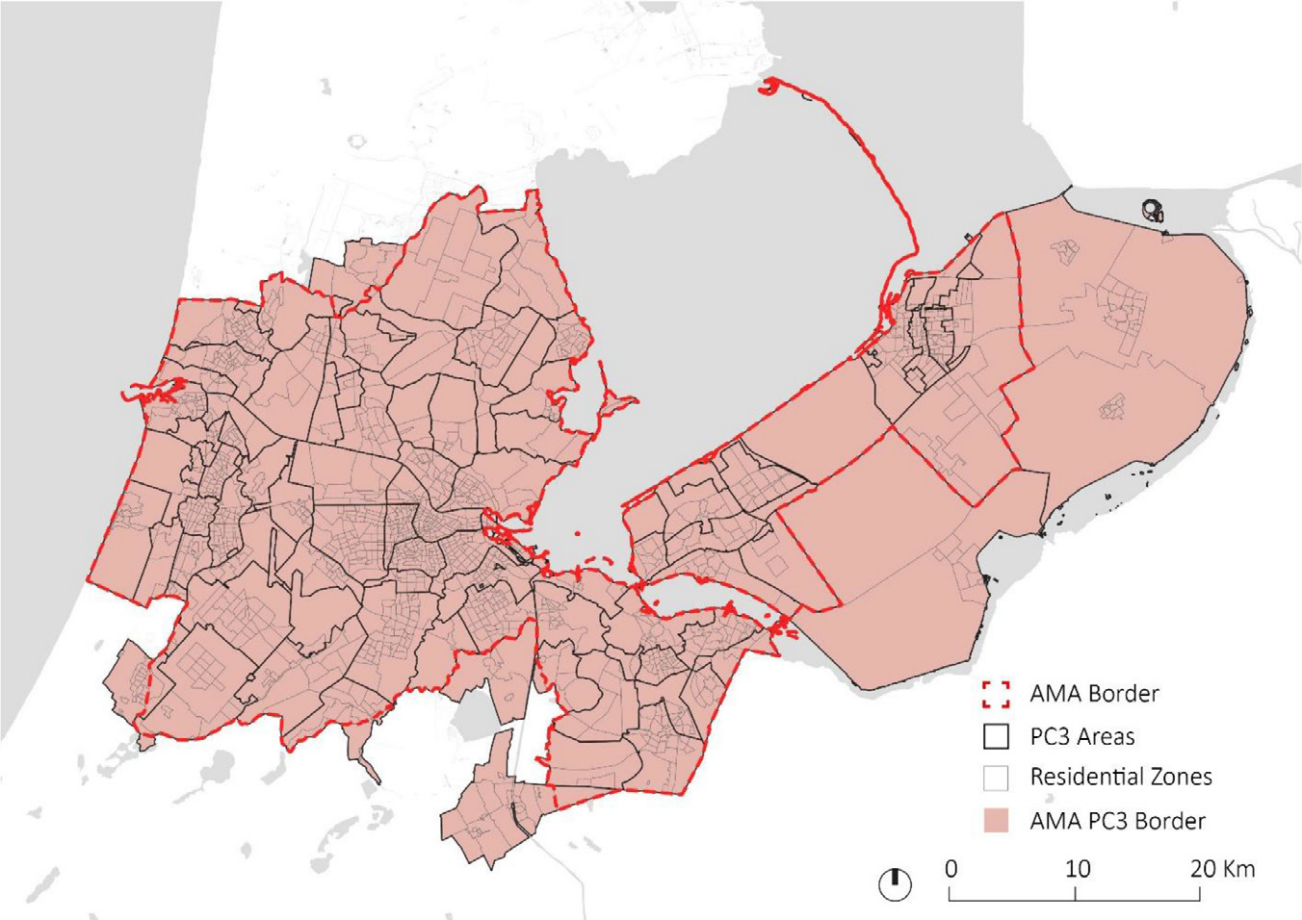
Dataset area. PC3s residential zones in the dataset area (light red), and the official border of AMA (intense red). (For interpretation of the references to color in this figure legend, the reader is referred to the web version of this article.).

The final dataset area includes 79 PC3 areas, 392 four-digit postcodes, and 1661 residential zones. The residential zones raw census data [5] is aggregated on PC3 scale, specifically the attributes on population number, number of households, number of vehicles, and number of companies (Fig. 3).

### 3.2 Network analysis

Two network analyses are carried out to generate spatial data for the final datasets. The Residential Parking Capacity calculation and the Road Radius analysis at the PC3 scale. The road network from Open Street Map was first prepared, creating three shapefiles (Table 2) with different road types, using the attribute “fclass”. The first shapefile includes only car-transit roads. The second shapefile includes only potential parking streets. The third one includes only walkable streets. Two assumptions on the types of streets are made:

- “Unclassified” (12.79% of total features) and “unknown” (0.01% of total features) roads are considered parking, car-transit, and walkable streets (Table 2).
- Only “residential”, “service”, “tertiary”, “unclassified”, and “unknown” roads are considered as potential residential parking streets (Table 2).

**Table 2:** Types of streets considered for the three road shapefiles. Source: the authors.

“fclass” attribute	Potential Residential Parking Streets	Car-Transit Roads	Walkable Streets
Bridleway	no	no	Yes
Cycleway	no	no	No
Footway	no	no	Yes
Living street	no	yes	Yes
Motorway	no	yes	No
Motorway_link	no	yes	No
Path	no	no	Yes
Pedestrian	no	no	Yes
Primary	no	yes	Yes
Primary_Link	no	yes	Yes
Residential	yes	yes	Yes
Secondary	no	yes	Yes
Secondary_Link	no	yes	Yes
Service	yes	yes	Yes
Steps	no	no	Yes
Tertiary	yes	yes	Yes
Tertiary_Link	yes	yes	Yes
Track	no	no	Yes
Track_grade	no	no	Yes
Trunk	no	no	Yes
Trunk_link	no	no	Yes
Unclassified	yes	yes	Yes
Unknown	yes	yes	Yes

#### 3.2.1 Parking capacity calculation

Parking capacity on roads and parking areas is calculated. Residential parking capacity is assumed to exclude roads that are not within walking distance of residential and mixed-used buildings.

For parking areas, the following operations are performed:

1. “gis_osm_traffic_a_free_1” GIS polygon shapefile from open street maps is used.
2. Car parking areas are selected using “fclass” equal to “parking”, “parking multistorey” and “parking underground”.
3. The number of parking lots for each parking area is calculated, assuming a circulation space of 33% and parking lots of 5.5 by 3 m.

**Fig. 4:**
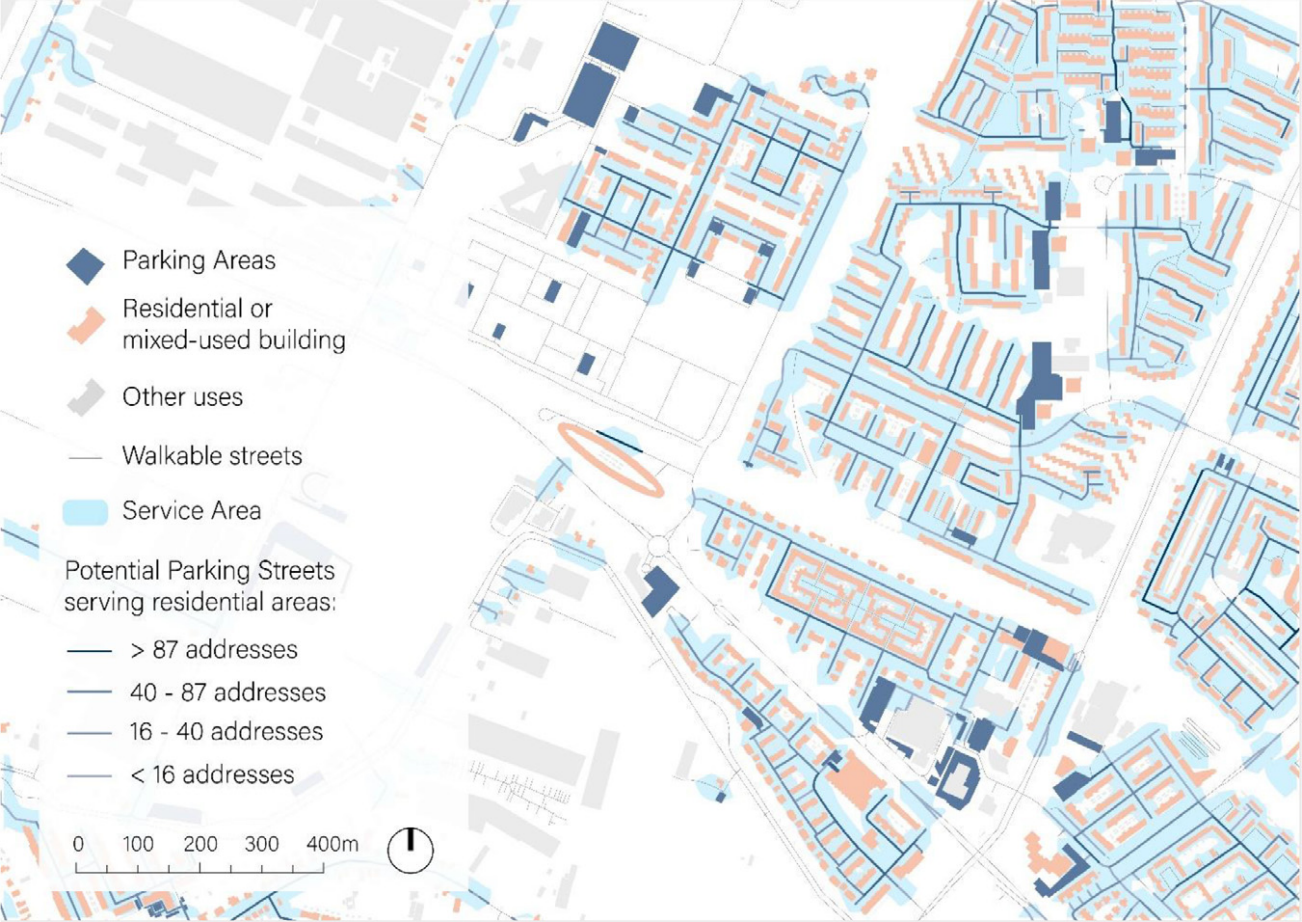
Parking streets zoom-in. Streets classified by number of addresses served according to legend.

For parking streets, the following operations are performed:

1. Residential and mixed-used buildings are selected from the raw cadaster dataset in the AMA. “Gebruiksfunctie = woonfunctie or overige gebruiksfunctie” (in English: “Use = Residential or Other Use”).
2. A spatial join between BAG 3D and BAG Adres GIS shapefiles is carried out to aggregate information on the number of addresses in each building polygon in the AMA.
3. The resulting polygon shapefile is transformed into a point database using the centroids of buildings’ polygons.
4. Using the GIS shapefile with walkable streets, a Service Area network analysis is carried out, considering a walking distance of 400m, and using the centroids of residentials and mixed-used buildings as origins (facilities). Service areas materialized as independent polygons (one per building centroid).
5. The potential parking streets are clipped (trimmed) using the Service Area.
6. It is assumed that “residential”-type streets, usually close to residential buildings, have one parking spot every 5 meters, whereas the other four types have one every 10 meters. The assumption is rather conservative, as intersections and parking-free areas are also considered in the calculation.
7. A spatial joint is performed between the “BAG adres” shapefile and the calculated Service Area to calculate the number of addresses inside the building of the Service Area. This information is added to the streets and used to calculate the number of addresses they may serve (Fig. 4).

Number of parking spots in parking areas and number of parking spots in streets are aggregated at the PC3 scale.

#### 3.2.2 PC3 radius calculation

The median road radius is calculated for each PC3, i.e., median distance from a PC3’s centroid to the road intersections located at its perimeter. Both the intersections and the road distance are calculated using the “car-transit road” shapefile.

The centroids are calculated according to PC3s’ geometry. The GIS polygon shapefile includes multi-part geometry. PC3s which include multiple non-adjacent polygons (parts) are separated into single parts. Centroids are calculated for every resulting polygon single part.

**Fig. 5:**
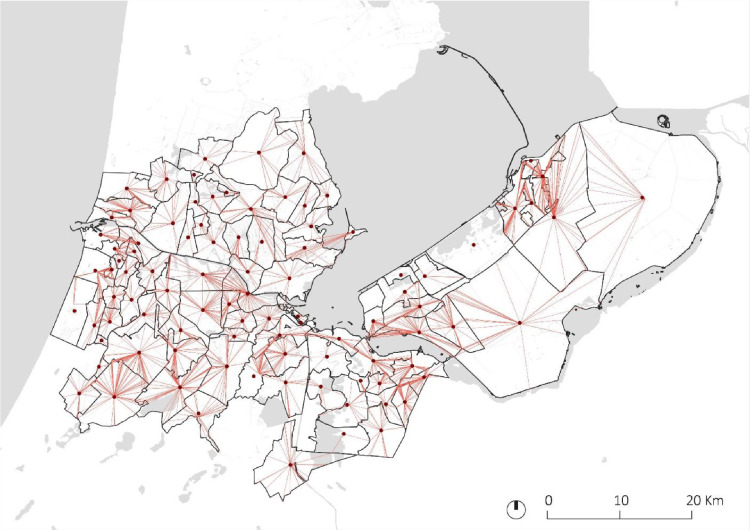
PC3 radius. PC3 radius (red) and centroids (dots) in the AMA. Radius are s geometric simplification of routes. See 3.2.2. (For interpretation of the references to color in this figure legend, the reader is referred to the web version of this article.).

Radius is calculated using an Origin-Destination analysis. First, the “car-transit road” shapefile is intersected with the PC3 polygon single parts borders, resulting in a GIS point shapefile. The intersection points hold information on the PC3 they originated from. The Origin-Destination analysis considers both distance and time. For the latter, the road average speed is considered (Table 1). The analysis results in a vector file with information on all routes between all origins and destinations. Routes that depart and arrive at the same PC3 single part are selected and extracted into a new vector shapefile (Fig. 5)^3^. Median radius road distance values are aggregated to the PC3 GIS polygon shapefile.

### 3.3 Electricity data calculation

The downloaded raw data included statistical data on the average energy demand per household and company [4] in kWh per year. Data is divided into gas and electricity supply per household and company for each postcode in the Netherlands. To integrate this data into the study datasets, five steps are followed:

- Data is aggregated at the PC3 scale.
- PC3s of the AMA are selected.
- Electricity supply per household is multiplied by total number of households [5] in each PC3 to obtain the total electricity supply for households per year (kWh per year) per PC3.
- Electricity demand per company is multiplied by total number of companies [5] in each PC3 to obtain total electricity demand for companies per year (kWh per year) per PC3.
- The two previous calculations are summed to obtain the total electricity demand per year (kWh) per PC3.

## 4 Aggregated Datasets

### 4.1 Shapefile with PC3 data

All data calculated in Section 2 is aggregated in the GIS polygon shapefile of PC3 areas in the AMA. The dataset is a combination of mobility, parking capacity, socioeconomic data, and electricity demand data. The SparkCity prediction model [8] foresees 3 million electric powered vehicles in the Netherlands by 2030. Based on this prediction, a projection of Electric Vehicles in each PC3 is also included in the aggregated dataset. Table 3 provides an overview of the data available in the final GIS dataset.

**Table 3:** Description of the data included in the GIS shapefile aggregated dataset.

Field name	Description	Unit	Source
PC3	Number of the 3-digit postcode	-	CBS
Park_St	Residential parking capacity in streets	Number of parking spots	See 3.2
Park_Ar	Parking capacity in parking areas	Number of parking spots	See 3.2
Park_Tot	Total parking capacity, including residential parking capacity in streets and in parking areas	Number of parking spots	See 3.2
kWh_Y2018	Total electricity supplied per year	kWh	See 3.3
Population	Total number of inhabitants	Number	CBS
Households	Total number of households	Number	CBS
Companies	Total number of companies	Number	CBS
Cars	Total number of cars	Number	CBS
EVs_2030	Projected number of Electric Vehicles by 2030	Number	Elaad, 2017
Rad_second	Median road distance from PC3 centroid to its perimeter. Expressed by time	Seconds	See 3.2
Rad_metres	Median road distance from PC3 centroid to its perimeter. Expressed by distance	Metres	See 3.2

### 4.2 Origin-destination analysis csv file

The second dataset includes Origin-Destination analysis with all possible combinations of PC3s as origin and destinations. The analysis, carried out using Arc GIS Pro software, considers all the PC3 centroids both as origins and destinations. Both the distance and the time spent are calculated for all routes. Time is calculated considering the average speed of roads based on their type (Table 1). The network used for the Origin-Destination analysis is the “car-transit road” (see 3.2). The results of the analysis are exported to a csv file. The radius results (both in time and distance) are added for every origin PC3. The final aggregated file is presented in Table 4.

**Table 4:** Description of the data included in the csv aggregated dataset.

Field name	Description	Unit
PC3_Orig	3-digit postcode where the trip originated from	Number
PC3_Dest	3-digit postcode where the trip ended	Number
Route	Origin and destination of the trip (origin-destination)	Name
Distance	Road distance from origin to destination	Meters
Time	Time spent from origin to destination according to roads’ maximum speeds	Seconds
Rad_med_m	Median metric distance from origin PC3 centroid to its perimeter, calculated as walking distance.	Meters
Rad_med_s	Median temporal distance from origin PC3 centroid to its perimeter, calculated as walking distance.	Seconds

## 5 Experimental Design, Materials and Methods

The dataset presented here brings several data sources together: (1) Spatial data, (2) electricity consumption data, (3) mobility data, (4) census data, and (5) cadaster data. All data is collected for the Amsterdam Metropolitan Area, a relevant metropolitan area in Europe, and was originally generated between 2017 and 2019. Several spatial analyses are performed with ArcGIS Pro software to aggregate all data, creating a GIS polygon shapefile with the data outcomes aggregated on three-digit postcode areas, supplemented with an Origin-Destination analysis. The five types of data were chosen as their combination and analysis bring opportunities to research on urban and regional mobility using GIS software, especially involving electric vehicles and electricity-based transport systems. The resulting dataset could be used for analyzing the potential location and distribution of electric vehicles charging infrastructure (similar to [9]), or in combination with mobility survey data to determine the impact to the electricity grid supply and infrastructure of new mobility systems (similar to [10] and [11]).

## Ethics Statements

The raw data of this study is provided by open, public GIS data sources, in full compliance with ethical requirements for publication in the journal of Data in Brief.

## CRediT authorship contribution statement

**Pablo Muñoz Unceta:** Conceptualization, Methodology, Software, Data curation, Writing – original draft, Visualization, Investigation, Validation, Writing – review & editing. **Bardia Mashhoodi:** Project administration, Conceptualization, Methodology, Writing – review & editing, Validation.

## Declaration of Competing Interest

The authors declare that they have no known competing financial interests or personal relationships that could have appeared to influence the work reported in this paper.

## Data Availability

Mobility, electricity, and parking data for EV infrastructure in the Amsterdam Metropolitan Area (Original data) (Mendeley Data).

## Footnotes

3 The resulting vectors of the Origin-Destination analysis represent the straight-line connecting origin and destination, but include distance and time considering the analysis network

## Footnotes

1 http://download.geofabrik.de/

2 The 130km/h limit applies only between 19:00 until 6:00. Outside this timeframe a new regulation from March 2020 sets the limit for high-speed motorways (“snelwegen”) in 100km/h to reduce nitrogen precipitation in nature.
